# The endemic and endangered Maugean Skate (*Zearaja maugeana*) exhibits short-term severe hypoxia tolerance

**DOI:** 10.1093/conphys/coz105

**Published:** 2020-01-18

**Authors:** Andrea J Morash, Jeremy M Lyle, Suzanne Currie, Justin D Bell, Kilian M Stehfest, Jayson M Semmens

**Affiliations:** 1 Institute for Marine and Antarctic Studies, University of Tasmania, 15-21 Nubeena Crescent, Taroona, Tasmania, Australia 7053, Australia; 2 Fisheries and Aquaculture Centre, Institute of Marine and Antarctic Studies, University of Tasmania, 15-21 Nubeena Crescent, Taroona, Tasmania, Australia 7053, Australia; 3 Department of Biology, Acadia University, 15 University Avenue PO Box 107 Wolfville, Nova Scotia, Canada B4P 2R6, Canada

**Keywords:** hypoxia, metabolism, environmental stress, anaerobic metabolism, endangered species

## Abstract

The endangered and range-restricted Maugean skate (*Zearaja maugeana*) is subjected to large environmental variability coupled with anthropogenic stressors in its endemic habitat, Macquarie Harbour, Tasmania. However, little is known about the basic biology/physiology of this skate, or how it may respond to future environmental challenges predicted from climate change and/or increases in human activities such as aquaculture. These skate live at a preferred depth of 5–15 m where the dissolved oxygen (DO) levels are moderate (~55% air saturation), but can be found in areas of the Harbour where DO can range from 100% saturation to anoxia. Given that the water at their preferred depth is already hypoxic, we sought to investigate their response to further decreases in DO that may arise from potential increases in anthropogenic stress. We measured oxygen consumption, haematological parameters, tissue–enzyme capacity and heat shock protein (HSP) levels in skate exposed to 55% dissolved O_2_ saturation (control) and 20% dissolved O_2_ saturation (hypoxic) for 48 h. We conclude that the Maugean skate appears to be an oxyconformer, with a decrease in the rate of O_2_ consumption with increasing hypoxia. Increases in blood glucose and lactate at 20% O_2_ suggest that skate are relying more on anaerobic metabolism to tolerate periods of very low oxygen. Despite these metabolic shifts, there was no difference in HSP70 levels between groups, suggesting this short-term exposure did not elicit a cellular stress response. The metabolic state of the skate suggests that low oxygen stress for longer periods of time (i.e. >48 h) may not be tolerable and could potentially result in loss of habitat or shifts in their preferred habitat. Given its endemic distribution and limited life-history information, it will be critical to understand its tolerance to environmental challenges to create robust conservation strategies.

## Teaser

The endangered Maugean skate is facing increasing environmental and anthropogenic stress that is decreasing DO in its preferred habitat. We found that it can withstand 48 h of severe hypoxia (20% air saturation) by significantly increasing anaerobic metabolism, but this may not be sustainable over longer periods of time.

## Introduction

Hypoxia occurs naturally in many aquatic ecosystems including coastal waters with high nutrient run-off, or systems characterized by strong density-driven stratification and limited mixing (Diaz and Rosenberg, 1995; Cloern, 2001). Both climate change and anthropogenic influences can further impact the severity or duration of hypoxia through increasing eutrophication of coastal waters and decreases in the solubility of oxygen due to increasing water temperature (Friedrich *et al.*, 2014). Indeed, the IPCC predicts that the frequency, severity and duration of hypoxic events will increase in the future (IPCC, 2014). Hypoxia is a serious threat to aquatic fauna and can limit viable habitat to hypoxia sensitive species. In fact, hypoxia can result in mass mortality or loss of biodiversity (Vaquer-Sunyer and Duarte, 2008). For range-restricted species, hypoxic events pose a severe threat as avoidance of hypoxic water in a restricted geographical space may be limited. Understanding how individual aquatic animals cope with hypoxic stress can help with conservation efforts and predicting future ecological impacts (Cooke *et al.*, 2012; Seebacher and Franklin, 2012).

A prime example of the impacts of anthropogenic induced hypoxia can be found in Macquarie Harbour, Tasmania; home to the endemic Maugean skate (*Zearaja maugeana*). Macquarie Harbour is ~280 km^2^ and is highly stratified; the surface layer is heavily tannined preventing light penetration much below the surface, is predominantly freshwater and exhibits seasonal thermal fluctuations. The middle layer is brackish with little thermal variation and reduced dissolved oxygen (DO), and the bottom layer is nearly marine with a salinity of 31 ppt, with little to no thermal variation, and in some areas, has limited oceanic input and minimal DO (0–20%) (Cresswell *et al.*, 1989). The saline stratification of Macquarie Harbour greatly reduces operating costs of salmonid aquaculture as the pathogen responsible for amoebic gill disease cannot survive in freshwater (Clark and Nowak, 1999). The Tasmanian salmonid aquaculture industry has been growing steadily with increasing interest in farming within Macquarie Harbour due to the aforementioned benefits. However, salmonid farming is well known to have impacts on surrounding water quality (Schenone *et al.*, 2011) and can decrease DO in the water surrounding the sites due to increased nutrient loading (Johansson *et al.*, 2006; Oppedal *et al.*, 2011; Burt *et al.*, 2012).

Little is known about the biology of the Maugean skate (*Zearaja maugeana*), which is listed as endangered under the Threatened Species Protection Act (Tasmania), the Environmental Protection and Biodiversity Conservation Act (Commonwealth) and is listed on the International Union for the Conservation of Nature Red List of Threatened Species (Last *et al.*, 2016). It is endemic to only two remote estuaries in western Tasmania, Australia; Macquarie Harbour and Bathurst Harbour (Last and Gledhill, 2007). However, the status of the Bathurst Harbour population is currently uncertain, with no individuals recorded from the area for over 20 years (Treloar *et al.*, 2017), whilst the population in Macquarie Harbour is believed to be ~ 3200 individuals (Bell *et al.*, 2016). As a member of the *Rajiformes* order, the Maugean skate is expected to have a conservative life history, reaching sexual maturity at a late age (Henderson *et al.*, 2004; Ebert, 2005; Matta and Gunderson, 2007; Colonello *et al.*, 2012). On the other hand, recent preliminary ageing data suggests that the Maugean skate may be short-lived for a rajiform and mature at a relatively young age (Bell *et al.*, 2016). It has been shown to have an asynchronous, discontinuous reproductive cycle (Bell *et al.*, 2016), however, fecundity has not yet been established. The relative lack of life-history data combined with its endemic distribution, low genetic diversity (Weltz *et al.*, 2018b) and limited diet (Weltz *et al.*, 2018a) provide challenging conditions for conservation of this species. In addition, throughout the last century Macquarie Harbour has seen increasing anthropogenic stress such as the damming of major tributaries for hydroelectricity, mining effluent, commercial/recreational fishing and salmonid aquaculture, which can potentially introduce several environmental stressors for this species. Based on acoustic telemetry, the preferred depth for the Maugean skate is between 5 and 15 m where ranges of DO are generally 30–80%, temperature from 12 to 15°C and salinity from ~18 to 24 ppt (Bell *et al.*, 2016). Some individuals have, however, been detected to depths of at least 55 m suggesting some level of tolerance to the wide array of environmental conditions here, including severe hypoxia (<10% DO)/anoxia.

To date, nothing is known about the hypoxia tolerance of the Maugean skate, or the strategies used to survive during hypoxic exposure. However, given its movement patterns through hypoxic zones in the Harbour, we expect the species can tolerate hypoxia. The metabolic costs associated with moving through hypoxic zones and the tolerable extent and duration are unknown. Other elasmobranch species are able to tolerate hypoxia/anoxia for short periods of time. The epaulette shark (*Hemiscyllium ocellatum*), and closely related grey carpet shark (*Chiloscyllium punctatum*), both tolerate periods of hypoxia and even 1.5 h of anoxia, but use divergent mechanisms to cope (Chapman and Renshaw, 2009). Epaulette sharks are cyclically exposed to severe hypoxia during nocturnal low tides, and respond by lowering their metabolic, ventilatory and heart rates, and upregulating anaerobic metabolism (Söderström *et al.*, 1999; Routley *et al.*, 2002). Grey carpet sharks have a varied distribution and are typically not exposed to the cyclic hypoxia on reef flats. However, they too can tolerate anoxia and respond with an increase in erythrocyte count, haematocrit and haemoglobin immediately following anoxia (Chapman and Renshaw, 2009), but no change in ventilation (Chapman *et al.*, 2011). The Maugean skate may rely on any combination of these mechanisms depending on the extent and exposure to hypoxia.

Since 2009 there has been a clear decline in DO in Macquarie Harbour (MHDOWG, 2014), and further decreases may limit suitable habitat for the Maugean skate and place additional stresses on this endangered species, in what may be its only remaining population stronghold. The objective of this study was to determine the hypoxia tolerance of the Maugean skate and to understand the biochemical and molecular mechanisms used to withstand hypoxic events. These data will give critical insight into how these endangered skate may respond to future hypoxic challenges, which may be more severe or longer in duration providing information necessary for conservation efforts.

## Methods

### Water chemistry

**Figure 1:**
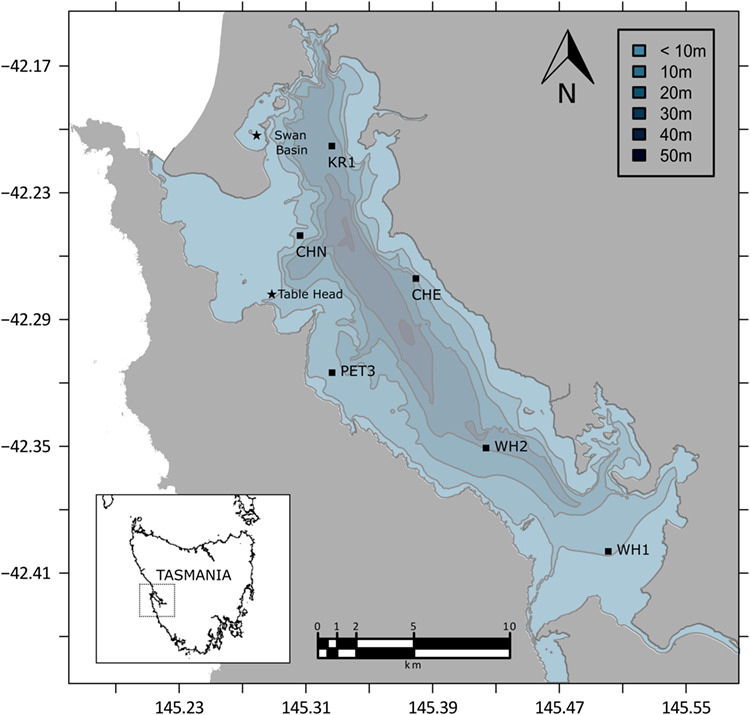
Map of Macquarie Harbour showing bathymetry and location of the environmental loggers (squares) and sites from which Maugean skate were sampled (stars). Insert shows map of Tasmania.

Monthly water column DO levels were measured continuously in Macquarie Harbour, Tasmania from 2014 to 2018 at six sites (Fig. 1) using a Sonde/CTD. The data were grouped into 5 m depth bins and the temporal trend smoothed with generalized additive models (GAM) using ggplot in R (v. 3.2.2).

### Animal collection and care

Twelve Maugean Skate (average mass = 1.72 ± 0.27 kg; 8 female, 4 male) were captured at 10 m depth by gillnet in the Table Head and Swan Basin regions of Macquarie Harbour, Tasmania (Fig. 1) during November of 2014. Gillnets were standard monofilament “graballs” (50 m long by 33 mesh drop, 114 mm stretched mesh), the type commonly used by recreational fishers. Gillnets were set during the morning hours and retrieved within 2–3 h to ensure that the skate were in good condition. Upon capture, skate were measured (total length), sexed and tagged with PIT tags and then placed in a 250 L tank with water that had been pumped from ~10 m depth to ensure water chemistry was consistent with the depth of capture for the majority of individuals. Within 2 h of capture the 12 skate were transported to an onshore laboratory and transferred to a 1200 L tank containing water pumped from 10 m depth from the area of capture. Oxygen saturation at this depth of the harbour is ~ 55% O_2_ and this was maintained in the holding tank and used as our control. The temperature of the holding tank was not regulated, and fluctuated with the daily atmospheric temperature between 15 and 18°C for the duration of the experiment. Water circulation within the tank was maintained by a large pump on the bottom and oxygen levels were monitored using a HQ40d DO metre and LDO probe (HACH) and maintained at 55% by regulated injection of either nitrogen or oxygen. Skate were acclimated to these conditions for 24 h prior to experimentation. Skate were not fed for the duration of the experiment to remove the effect of the energetic cost associated with digestion (i.e. specific dynamic action) during respirometry (see review by Clark *et al.*, 2013).

All procedures were undertaken with the approval of the University of Tasmania Animal Ethics Committee (permit A13468), scientific permits 13 125 and 14 139 issued under Section 14 of the Living Marine Resource Management Act 1995 and permits TFA 13 982, 14 019 and 14 253 issued under Regulation 4 of the Threatened Species Protection Regulations 2006 and Section 29 of the Nature Conservation Act 2002. These permits covered the capture, possession and biological sampling of an endangered species, the deployment of research fishing gear and the deployment of moorings within the Macquarie Harbour, including the World Heritage Area.

### Experimental protocol

After the 24-h acclimation period, half of the skate were transferred to a separate 1200 L experimental tank containing the same water as the control tank but maintained at an oxygen saturation of 20% O_2_, representative of areas of low oxygen saturation in the harbour. DO was maintained at 20% by monitoring O_2_ content and bubbling in N_2_ as required. Both control (55% O_2_; three male, three female; average mass 1.63 ± 0.4 kg) and treatment (20% O_2_; one male, five female; average mass 1.82 ± 0.4 kg) groups were held at their respective DO levels for a total of 48 hours. After the first 24 h, a 0.5–1 mL blood sample was taken from the caudal vasculature immediately posterior to the cloaca with a 22-gauge heparinized syringe prior to the start of metabolic measurements. This process took ~ 1 min per animal. Each skate (fasted for 48 h) was then transferred to an individual respirometer to determine their metabolic rate across the full range of oxygen saturation (75–20%; see below). A second blood sample was taken at the completion of the respirometry tests and the skate were returned to their respective tanks and allowed to recover for 24 h. A third caudal blood sample was taken prior to skate being euthanized via cranial puncture. Heart, liver, rectal gland, gill and white muscle (WM) tissue were dissected, weighed and frozen immediately in liquid nitrogen for later metabolite and enzymatic analysis.

### Haematological analysis

Haematocrit, haemoglobin, glucose and lactate were measured in whole blood immediately after each blood sample was taken. Haematocrit was measured in duplicate in a SpinCrit Microhaematocrit centrifuge (SpinCrit). Haemoglobin was measured using HemoCue Hb210+ system in accordance with the manufacturer’s instructions and values were corrected according to (Clark *et al.*, 2013) for fish. The mean cell haemoglobin concentration (MCHC), a measure of the mean haemoglobin within each red blood cell, was calculated by dividing the corrected haemoglobin values by the haematocrit values. Whole blood glucose and lactate were measured using hand held OneTouch Ultra2 glucometer (LifeScan, Milpitas, California) and a Lactate Pro™ (Arkray Global Business, Inc.), respectively. The remaining blood samples were spun at 13 000 rpm for 4 min to separate red blood cells and plasma. The plasma was separated into a clean microcentrifuge tube, the buffy coat removed from the remaining red blood cells and both fractions were stored at −80°C until further analysis.

### Respirometry

Respirometry measurements were conducted in large flat plastic trays appropriate for the size of the skate to ensure they remained still. Each skate was transferred to an individual tray containing water from their respective tanks and a small pump to maintain water circulation, and allowed to rest for 1 h prior to the start of the measurements. At the start of the trial, O_2_ saturation was raised to ~75% and the trays were then sealed with gas impermeable, translucent plastic sheets (previously tested for 24 h over a 0% O_2_ tank with no oxygen transfer). Oxygen consumption was recorded using a Fibox O_2_ probe (PreSens Fibox) throughout the duration of the trial. The O_2_ level was allowed to drop as the skate consumed the oxygen from 75 to 20% (~1–2 h), which represents the typical range of oxygen saturation that the skate are found in. Below 20% DO, O_2_ consumption was negligible, and the trials were concluded. The average slope of O_2_ consumption from each 5% increment was used to calculate routine aerobic metabolic rate (ṀO_2_; mgO_2_·kg^−1^·h^−1^), considering background O_2_ consumption rate (measured as oxygen depletion in the empty respirometer), the volume of the tray, skate mass, temperature and barometric pressure. The values for each skate at each DO increment were then averaged.

### Tissue assays

We used heart, liver and WM tissue to assess the metabolic state of the skate. We used tissue glycogen as an indicator of energy storage (Enes *et al.*, 2009) that could be used for anaerobic energy production, tissue lactate as an indicator of anaerobic metabolism (Milligan and Wood, 1986), citrate synthase and lactate dehydrogenase as indicators of aerobic and anaerobic metabolism, respectively (Somero and Childress, 1985). We also measured heat shock protein HSP70 in all five tissues collected as a measure of hypoxia-induced cellular stress (Currie and Tufts, 1997).

Frozen tissues were powdered under liquid N_2_ using a mortar and pestle and kept at −80°C until further analysis. For citrate synthase and lactate dehydrogenase, powdered tissue was homogenized in 20x w:v of ice cold homogenisation buffer (100 mM potassium phosphate, 5 mM EDTA and 0.1% Triton x-100 at pH 7.2). Tissue lactate and glycogen were isolated as described below. All assays were performed in triplicate at room temperature in 96 well clear bottom plates using a SpectraMax Plus 384 spectrophotometer (Molecular Devices, Sunnyvale, CA), and we collected data using Softmax Pro 4.7.1 software (Molecular Devices, Sunnyvale, CA).

*Citrate Synthase (CS).* CS was measured according to (Heinrich *et al.*, 2014). The CS assay buffer contained 20 mM Tris (pH 8.0), 0.1 mM 5,5-dithiobis (2-nitrobenzoic acid) and 0.3 mM acetyl-CoA. The reaction was initiated by the addition of 0.5 mm oxaloacetate, and absorbance was measured for 5 min at 412 nm. Control samples were assayed without oxaloacetate to control for background hydrolase activity.

*Lactate Dehydrogenase (LDH).* LDH was measured using a modified assay from (Johnston *et al.*, 1977). The assay buffer consisted of 50 mM TRIS-HCl, 2 mM sodium pyruvate and 0.15 mM NADH. Absorbance was measured at 340 nm for 3 min.

*Tissue Lactate*. Tissue lactate was extracted and measured as in Tunnah *et al.* (2016a). Powdered tissues were homogenized in 1:4 w:v 8% PCA containing 1 mM EDTA using a PowerGen 125 homogenizer (Fisher Scientific, Ottawa, Canada). Samples were centrifuged at 16 438 x g for 4 min at 4°C. The supernatant was neutralized with 2 mM KOH containing 0.4 mM imidazole. Samples were centrifuged again at 16 438 x g for 1 min at 4°C. The final supernatant was used for lactate quantification relative to an L-lactic acid standard curve. The assay buffer contained 0.16 M glycine, 0.13 M hydrazine, 1.9 M NAD^+^ and 10 U lactate dehydrogenase. The reaction was run to completion at room temperature (~35 min) and absorbance was measured at 340 nm.

*Tissue Glycogen*. Tissue glycogen was extracted and fully hydrolysed to glucose according to (Clow *et al.*, 2004) and the hydrolysates were kept at −80°C until further analysis. Glucose was measured according to (Bergmeyer *et al*., 1974) using a glucose standard curve. The assay media contained 250 mM imidazole, 5 mM MgSO^4^, 10 mM ATP, 0.8 mM NADP^+^. G6PDH (25 μL of 10 U/mL) was added to the wells and incubated at room temperature for 10 min to eliminate any endogenous glucose-6-phosphate. The plate was read at 340 nm to establish background absorbance before hexokinase (25 μL of 10 U/mL) was added to each well and incubated for 25 min. Absorbance was read again at 340 nm and [glucose] was calculated according to the standard curve.

*HSP70*. All five tissues collected were ground in liquid nitrogen to a fine powder, and a 15x weight-to-volume ratio was used to dilute the samples in 1x homogenisation buffer (50 mM Tris Base, 70 mM SDS) with the addition of 1X Protease Inhibitor Cocktail (PIC004.1, Bioshop Canada). We used a PowerGen125 homogenizer at 50% power to subject the samples to 20 s bursts, and then spun solutions at 14 800 x g for 10 min. We assayed the supernatants for soluble protein concentration using the detergent-compatible DC assay against a BSA standard (Bio-Rad). Protein extracts were prepared for electrophoresis based on equivalent total protein content (15 μg/well) using 1x sample buffer (Life Technologies) and 50 mM DTT, then heated 5 min at 70**°**C. We separated proteins in a Bolt 4–12% Bis Tris SDS-PAGE gel (Life Technologies) with 200 V power for 34 min, then transferred to polyvinylidene difluoride (PVDF, Bio-Rad) membrane for 60 min at 30 V. Each gel had a three-point serial dilution of a reference fish to give a standard curve for comparison. We blocked membranes overnight at 4**°**C in 5% _w/v_ fat-free milk powder dissolved in TBS-T (Tris, 20 mM; NaCl, 137 mM; Tween-20, 0.1%_v/v_), then incubated in 1:10 000 rabbit anti-HSP70 antibody for 1 h (Agrisera AS05_083A; recognizes both constitutive and inducible isoforms) and finally in 1:20 000 goat anti-rabbit IgG HRP conjugated antibody (Abcam ab6721) for 1 h. Membranes were rinsed with TBS-T solution five times after each antibody incubation. Chemi-luminescent images were obtained using ECL Select reagent (GE Healthcare) and a VersaDoc™MP 400 System (Bio-Rad). Band densities for samples were determined against the standard curve using the ImageLab software (v 4.0, Bio-Rad).

*Protein*. We measured tissue soluble protein using a DC Protein Assay kit (Bio-Rad) using bovine serum albumin as a standard.

### Statistical analysis

We used R (version 3.6.0, R Core Team 2019) statistical software for our analysis. To determine whether acclimation to low DO had an impact on metabolic response to acute hypoxia, we compared two linear mixed models (R package lme4) of the relationship between metabolic rate and ambient DO, one with both ambient DO and acclimation treatment (hypoxic or normoxic; Model 1) and one with only ambient DO included as the predictor variable (Model 2). Individual ID was included as a random effect in both models to account for repeated metabolic rate measurements for each individual. Models were compared using the Akaike Information Criterion (AIC), the significance of parameters (*α* = 0.05) of each of the models was estimated using the Wald Chi-squared test. Visual inspection of model residuals was used to ensure assumptions of homoscedasticity and normality of residuals were not violated. Models were fitted in the R software environment for statistical computing (R Core Team, 2014). A repeated-measures two-way ANOVA and Holm-Sidak *post-hoc* tests were used to test for differences in haematological factors between treatments and over time. *T*-tests were used to test for differences in tissue enzymes, metabolites and HSP70 expression between the control and hypoxic groups. *P* ≤ 0.05 was considered to be statistically significant.

## Results

### Environmental conditions

**Figure 2:**
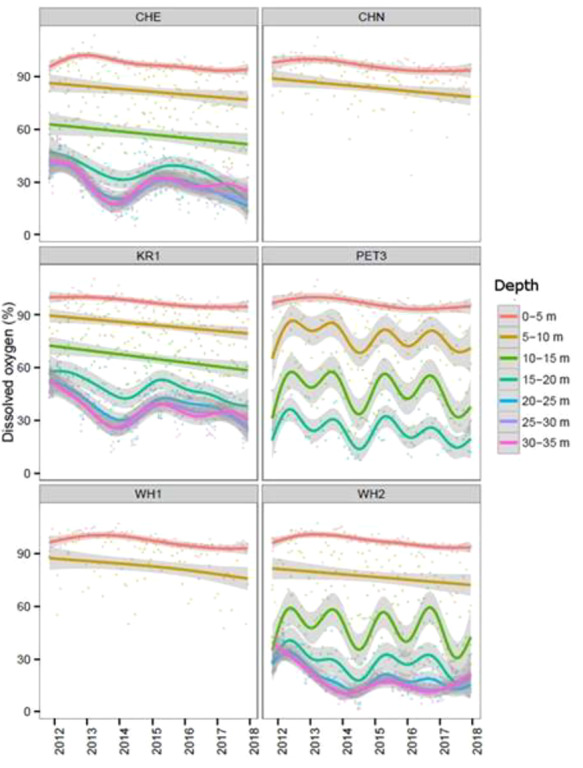
Water column DO levels measured continuously in Macquarie Harbour from 2014 to 2018 at six sites (see Fig. 1 for site code locations). The DO data are grouped into 5 m depth bins and fitted with a GAM.

DO ranged from 90 to 30% between 5 and 15 m, the preferred depth rage of the Maugean skate (Fig. 2). Over the 6-year environmental data collection period, there is a downward trend in the DO of Macquarie Harbour at all six sampling locations (Fig. 1).

### Respirometry

**Figure 3:**
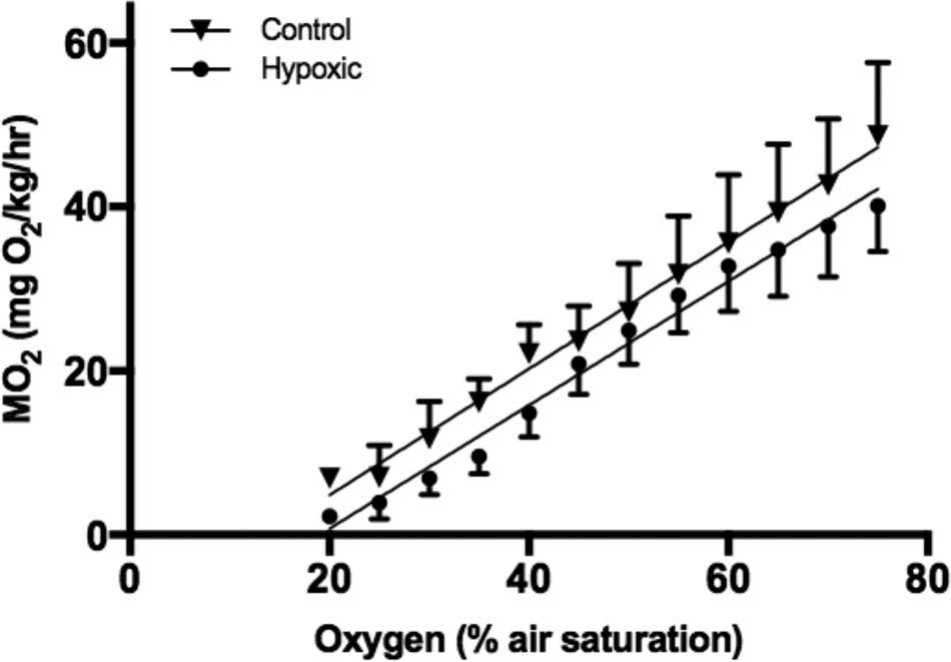
Rate of O_2_ consumption (MO_2_) across a range of environmental DO concentrations of skate acclimated for 24 h to either control (55% DO) or hypoxic (20% DO) conditions. Each point represents the same six individuals for each treatment group and are presented with ±SE for clarity. Oxygen was allowed to decline naturally, as it was consumed by the skate.

During the experimental trials, the MO_2_ declined linearly in both control and hypoxic groups as the ambient oxygen declined (Fig. 3). Treatment did not have any effect on MO_2_, as linear mixed model results showed that inclusion of acclimation treatment as a fixed effect did not result in a significant reduction of the AIC (ΔAIC<2) and the acclimation parameter was not significant in the full model (Model 1; Table 1).

**Table 1:** Comparison of linear mixed models with and without acclimation treatment as a fixed effect

Model	AIC	Parameter	df	*F*-value	*P*
Model 1: MO^2^~Ambient DO + Acclimation	961.1874	Ambient DO	118	393.633	<0.001
		Acclimation	10	1.525	0.245
Model 2: MO^2^~Ambient DO	961.4840	Ambient DO	118	393.647	<0.001

### Haematology

Despite differences in oxygen consumption, there was no significant difference in haemoglobin or haematocrit between groups or after 48 h in their respective treatment groups (Table 1). However, the MCHC was significantly lower in the hypoxic group (Table 2; *P* = 0.005) at 24 and 48 h. Whole blood lactate, a measure of anaerobic activity, was not detectable in the control group at either 24 or 48 h and was significantly higher in the hypoxic group at both time points (Table 2; *P* = 0.003). There was a significant effect of time (*P* = 0.0068), but not oxygen, on the whole blood glucose concentration. Whole blood glucose declined over time in both control and hypoxic groups (Table 2).

**Table 2:** Whole blood haemoglobin, haematocrit, MCHC, glucose and lactate of skate under control (55% DO) or hypoxic (20% DO) conditions for 24 and 48 h. Data are presented as mean ± S.E. *n* = 6 for all measurements

	*T* = 24		*T* = 48 h	
	Control	Hypoxic	Control	Hypoxic
Haemoglobin (g/L)	30.8 ± 2.9	23.1 ± 3.0	30.6 ± 1.9	23.1 ± 3.6
Haematocrit (%)	15.2 ± 1.1	17.2 ± 2.0	16.7 ± 1.6	18.5 ± 2.8
MCHC	203 ± 8.5	135.7 ± 13.2^a^	188 ± 10.4	132.3 ± 19.8^a^
Glucose (mmol/L)	1.7 ± 0.2	2.4 ± 0.3^A^	1.1 ± 0.3	1.3 ± 0.3^B^
Lactate (mmol/L)	ND	7.8 ± 1.6^a^	ND	9.4 ± 1.9^a^

^a^Significant effect of O_2_.

Uppercase letters show significant effect of time.

MCHC, mean corpuscular hemoglobin concentration; ND = not detectable.

### Metabolites

**Figure 4:**
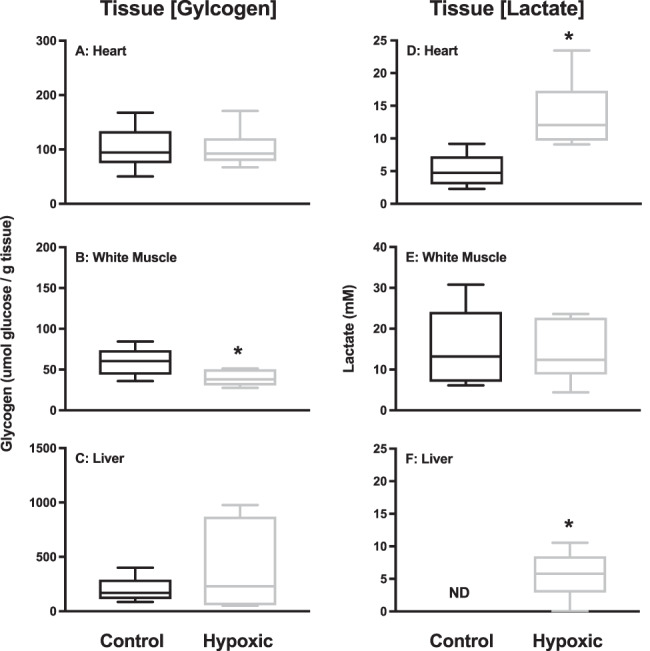
Concentration of glycogen (A-C) and lactate (D-F) in heart (A&D), white muscle (B&E) and liver (C&F) after 48 h in control (55% DO) or hypoxic (20% DO) conditions. Values are mean ± SE. *n* = 6 for each tissue and treatment. Asterisk denotes significant difference (*P* < 0.05).

**Figure 5:**
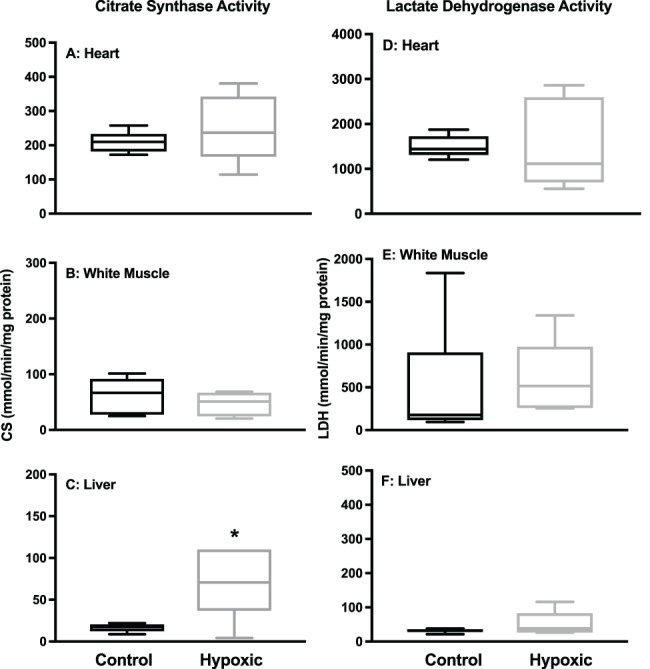
Activity of citrate synthase (A-C) and lactate dehydrogenase (D-F) in heart (A&D), white muscle (B&E) and liver (C&F) after 48 h in control (55% DO) or hypoxic (20% DO) conditions. Values are mean ± SE. *n* = 6 for each tissue and treatment. Asterisk denotes significant difference (*P* < 0.05).

We measured tissue lactate and glycogen (as glucose) in the heart, WM and liver to further understand the metabolic effects of hypoxia. We found that the concentration of glycogen did not differ between treatments in the heart or liver, but was significantly lower in the WM of the hypoxic group (Fig. 4B; *P* = 0.0232). In contrast, the concentration of lactate did not differ between treatments in the WM but was significantly higher in the heart (*P* = 0.0026) and liver (*P* = 0.0019) of the hypoxic group (Fig. 4D and F, respectively). Despite changes in blood and tissue lactate, tissue LDH did not change in any of the tested tissues between control and hypoxic skate (Fig. 5D–F). We measured CS as a proxy for aerobic capacity and found that there was no change in the heart or muscle, but a significant increase in CS activity in the liver of the hypoxic group (Fig. 5C; *P* = 0.0091).

**Figure 6:**
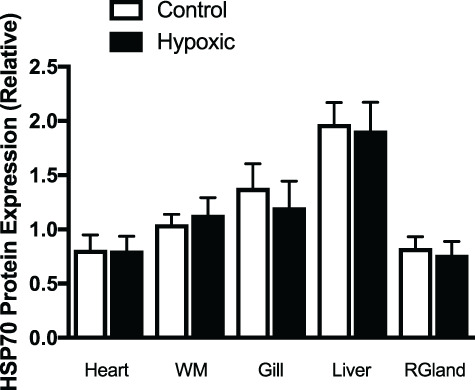
HSP70 in skate heart, white muscle (WM), gill, liver and rectal gland (RGland) after 48 hours in control (55% DO) or hypoxic (20% DO) conditions. Values are mean ± S.E. n = 6 for each tissue and treatment.

### HSP70

We measured HSP70 protein in heart, white muscle, gill, liver and rectal gland to determine if there were tissue-specific, hypoxic-induced stress responses and found no significant differences in HSP70 protein expression between control and hypoxic groups (Fig. 6).

## Discussion

Conservation efforts for endangered range-restricted species require comprehensive knowledge of the biology of the animals and the environmental conditions in which they are found, particularly when their environment is subject to anthropogenic impact. For the Maugean skate, there has been a strong focus on describing the environmental conditions of Macquarie Harbour, which are characterized by low DO bottom waters (Cresswell *et al.*, 1989), and how these conditions are degrading under anthropogenic pressures (Ross *et al.*, 2016; Ross and Macleod, 2017a, 2017b). Until relatively recently, there had been limited investigation into their biology. However, the recent decline in DO in Macquarie harbour (MHDOWG, 2014; Ross and Macleod, 2016) has prompted numerous research endeavours to understand the biology of the endangered Maugean skate, and the effects of environmental disturbances on the population (Last and Gledhill, 2007; Bell *et al.*, 2016; Treloar *et al.*, 2017; Weltz *et al.*, 2018a, 2018b). This study is the first to describe the metabolic characteristics of the Maugean skate and its physiological response to environmentally relevant hypoxia.

The Maugean skate appears to be an oxyconformer (*sensu*Dejours, 1981) as indicated by the linear decline in the rate of oxygen uptake in both control and hypoxic groups (Fig. 3). Hypoxia tolerance in elasmobranchs ranges from species capable of surviving extremely low levels of DO (<2 kPa; electric ray (*Torpedo marmorata*); Hughes and Johnston, 1978) and even short periods of anoxia (Epaulette shark (*Hemiscyllium ocellatum*); Renshaw *et al.*, 2002) to the relatively hypoxia-sensitive species (Eastern shovelnose ray (*Aptychotrema rostrata*); Speers-Roesch *et al.*, 2012. Many hypoxia tolerant animals exhibit a greater ability to extract O_2_ from the water, even as the environmental O_2_ concentration drops (i.e. oxyregulator). These physiological mechanisms include increases in ventilation, circulation and haemoglobin concentration for efficient transport of oxygen, or changes at the cellular level for diffusion into the tissues. However, beyond a certain concentration (*P*_crit_; critical oxygen tension), the rate of O_2_ consumption can no longer be maintained, and drops along with the environmental O_2_ concentration (oxygen conformity). Some species show relatively little regulation of O_2_ uptake as environmental O_2_ declines, and are known as oxyconformers. Often, there is a correlation between an animal’s ability to oxyregulate and its environment; hypoxia tolerant species tend to live in environments where hypoxic events are prevalent, and have developed coping mechanisms suited to this environment. Whether animals regulate or conform, when the rate of O_2_ consumption declines and tissues become hypoxic, there are two main mechanisms employed by most vertebrate animals for survival; (1) rely on O_2_-independent mechanisms of energy production (i.e. anaerobic glycolysis), or (2) reduce energetically costly processes (metabolic rate depression) to balance energy supply and demand (Richards, 2009). These mechanisms are largely dependent on the extent and duration of the hypoxic event and can vary between species.

The metabolic rate and rate of decline were similar between groups at all environmental levels of oxygen suggesting that metabolic rate depression is not a mechanism used by these animals when exposed to short term hypoxia. Active metabolic rate depression is common amongst hypoxia/anoxia tolerant animals (Van Waversveld *et al.*, 1989; Buck *et al.*, 1993; Hochachka *et al.*, 1996; Guppy and Withers, 1999; Nilsson and Renshaw, 2004; Storey and Storey, 2004; Jibb and Richards, 2008) to match oxygen supply and demand. The DO within the preferred habitat of the Maugean skate is low (~55% DO), and these animals have been detected on many occasions in various parts of the harbour where the DO is extremely low (<20% DO). Given the frequent exposure to these conditions, 48 h of 20% dissolved O_2_ may not have been challenging enough to elicit metabolic rate depression in this study.

Lactate, a major marker of anaerobic metabolism, was not detectable in the blood of the skate at 55% DO suggesting that even at this oxygen level they are able to supply their energetic needs through aerobic metabolism. Increases in whole blood and tissue lactate, and decreases in glycogen stores at 20% DO, however, suggest that these skate are relying more heavily on anaerobic metabolism at oxygen levels lower than those of their preferred habitat. Similar to the epaulette shark response to hypoxia/anoxia, we found a decrease in MCHC, which is suggestive of cell swelling. In teleosts, this has been attributed to a decrease in Na^+^/K^+^ ATPase activity when oxygen in decreased, or there is a change in extracellular pH (Fievet *et al.*, 1987; Salama and Nikinmaa, 1990), but has not been investigated in anoxia-tolerant elasmobranch species. In the short-term, it appears that this hypoxic exposure is not a major challenge to skate, and that anaerobic metabolism was sufficient for their energy requirements. This is supported by a lack of increase in HSP70 expression in any tissues tested after 48 h of 20% DO. HSPs are a conserved group of constitutive and inducible proteins, which act to protect against protein mis-folding in cells exposed to a variety of stresses such as hypoxia, oxidative stress, ATP depletion or high temperature. HSP induction in elasmobranch species has not been well studied, but anoxia (Renshaw *et al.*, 2004) and osmotic challenges (MacLellan *et al.*, 2015; Morash *et al.*, 2016; Tunnah *et al.*, 2016b) will induce HSP70 in a species- and tissue-specific manner in some species of sharks. In the anoxia tolerant epaulette shark for example, HSP70 expression was only induced after anoxic stress, but not hypoxic (5% of normoxia) stress. Renshaw *et al*. (2004) suggest that in this hypoxia tolerant species, the “damage threshold” was not exceeded to stimulate HSP70 expression. Several researchers have suggested that there are both O_2_ and metabolic sensors (i.e. decreased ATP), which may activate HSP70 expression during hypoxia (Currie *et al.*, 1999; Lutz and Prentice, 2002). For the Maugean skate, the level of hypoxia may not have been sufficient enough to activate either an O_2_ or a metabolic challenge for the induction of HSP70.

Although the effects of hypoxia have not been investigated broadly in skate species, it has been shown that hypoxia can influence the distribution and abundance of other shark and ray species, particularly in estuarine ecosystems (Schlaff *et al.*, 2014). Species-specific hypoxia tolerance and strategies for coping with hypoxia are variable, but highlight that many species (at least those tested) are tolerant to short-term exposure to hypoxia. Several species have been investigated in the shallow seagrass meadows of the Gulf of Mexico, an area that experiences seasonal hypoxia. The obligate ram-ventilators, bonnethead shark (*Sphyrna tiburo*), blacknose shark (*Carcharhinus acronotus*) inhabiting this environment increase swimming speed and mouth gape to increase oxygen consumption when exposed to hypoxia. In contrast, the buccal-ventilator, the Florida smoothound shark (*Mustelus norrisi*) shows a decrease in activity and metabolic rate (Carlson and Parsons, 2001). Atlantic stingrays (*Dasyatis sabina*), residents of shallow (<1 m) sea-grass beds between Chesapeake Bay and the Gulf of Mexico, exposed to daily 7 h hypoxia treatments of 30% DO for 20 days significantly increased their branchial surface area, but did not have any differences in haematocrit or haemoglobin (Dabruzzi and Bennett, 2014). Similarly, repeated exposure to hypoxia has been shown to confer an increase in hypoxia tolerance in the anoxia tolerant epaulette shark (*Hemiscyllium ocellatum*) (Routley *et al.*, 2002). The cownose ray (*Rhinoptera bonasus*) has been frequently tracked in similarly hypoxic environments in the Louisiana shelf of the Gulf of Mexico, suggesting it is also tolerant of hypoxia (Craig *et al.*, 2010). These studies collectively suggest that the ability to cope with hypoxia confers an adaptive advantage for these species, in that they can forage for longer in areas unexploited by predators or competitors. In these instances, hypoxic exposures are limited to short periods, or daily exposures with recovery periods in between. To our knowledge, our study is the first to investigate the physiological effects of prolonged hypoxia. The response of the Maugean skate to 48 h of constant hypoxia seems consistent with other hypoxia tolerant elasmobranch species; physiological alterations to cope with the hypoxia, but limited indication of overall stress. As benthic foragers, these skate would routinely be exposed to the low oxygen environments within Macquarie Harbour that may pre-condition them to surviving longer periods in hypoxia. In addition, sex may also play a role in physiological mechanisms of hypoxia tolerance. Sex was not a factor in selection for our treatment groups, and was skewed towards females in the hypoxia group. Although only tested once in *in vitro* brainstem preparations in frogs (Rousseau *et al.*, 2016), and in several studies of mammalian brains (von Arnim *et al.*, 2002; Kitano *et al.*, 2007; Wang *et al.*, 2008), sex may play a role in the underlying mechanisms associated with hypoxia tolerance, but whole animal *in vivo* hypoxia tolerance has not been established between sexes.

From 2009 to 2014, there was a clear decline in DO below 15 m, but values appear to have stabilized at ~30% DO from 2014 to 2018, down from ~ 55% DO pre-2009 (Ross and Macleod, 2017a). The decline in DO within the Harbour corresponded with historically low river flow as well as the expansion of salmonid aquaculture (MHDOWG, 2014). The naturally low levels of oxygen in the deep waters of Macquarie Harbour are a result of the significant stratification. However, other factors such as river flow (natural changes due to weather, or anthropogenic changes due to damming), weather conditions (i.e. storm events that facilitate vertical mixing) and biological oxygen demand resulting from nutrification and the aquaculture fish biomass near aquaculture sites, also influence the total DO throughout the Harbour. At present, it is unclear what effect DO has on the habitat range of the Maugean skate. Whilst they appear to tolerate significant declines in DO for at least 48 h, as shown here, it is unknown if they could tolerate this level of DO for extended periods of time. However, given the significant increase in tissue and blood lactate after 48 h of 20% DO, it is unlikely that the skate would be able to survive more than a few days in this condition. Lactate buildup can result in acidosis of blood and tissues due to limited buffering capacity in elasmobranchs (Dickson *et al.*, 1993; Ballantyne, 1997) and anaerobic metabolism is restricted by limited stores of glucose or gluconeogenic substrates (Ballantyne, 1997). This anaerobic state can be further exacerbated by recreational fisheries within the Harbour. Maugean Skate are common bycatch in recreational gillnets, and this type of catch and release has been shown in bull (*Carcharhinus leucas*) and bonnethead sharks to elicit metabolic acidosis (Hyatt *et al.*, 2018). It is also possible, given the stratification of the Harbour, for Maugean skate to be restrained in unfavourable DO conditions when caught on the gillnet that may further stress these individuals. Further experiments would be required to fully assess the long-term physiological effects of combined hypoxia and catch and release on this species.

Should hypoxic exposure persist beyond 48 h within the preferred depth of the skate, it is possible that they may be forced to spend increasing amounts of time at shallower depths where DO is higher. Whilst DO may be higher at shallower depths, salinity is lower. At present, the osmoregulatory capabilities of this species are unknown. They have been detected at depths <5 m, particularly at night, most likely foraging, suggesting that they can tolerate low salinity, or even fresh water for short periods of time (Bell *et al.*, 2016). However, <5% of elasmobranch species live in freshwater habitats due to a combination of physiological and biochemical issues (Ballantyne and Fraser, 2013), and thus it is likely that prolonged exposure to decreased salinity would have a negative effects on this species. More information on the osmoregulatory capacity of this species will be required to fully understand the effects of salinity on their depth utilisation.

Climate change is causing wide-spread effects on estuaries and coastal regions worldwide (Day *et al.*, 2008). In particular, severe bouts of hypoxia are increasing due to anthropogenic nutrification and/or up-welling of nutrient-rich hypoxic waters (Fennel and Testa, 2019). The conditions used here are not limited to the Maugean skate. Many elasmobranch species rely on estuaries for pupping and juveniles may spend considerable time in estuaries to avoid predation (Parsons and Offmayer, 2005; Heupel *et al.*, 2007). Understanding how elasmobranchs respond to hypoxic events will be critical for the conservation of many threatened species, especially given the fact that many elasmobranchs are slow to mature and/or reproduce (Compagno, 1990). Given the benefits for salmonid farming in Macquarie Harbour, it is likely that future requests to expand operations are imminent. However, further declines in DO as a potential result of expanded aquaculture may prove to be detrimental to the Maugean skate and other resident and/or transient species.

In conclusion, the Maugean skate can tolerate extreme short-term hypoxia by relying increasingly on anaerobic metabolism for energy production. This metabolic state does not appear to elicit a major cellular stress response and is likely a result of chronic exposure to moderate hypoxia in their preferred habitat within the Harbour. In the short-term, severe hypoxia may have negligible effects on the Maugean skate; however, chronic extreme hypoxia, whether a result of natural or anthropogenic sources, could potentially be detrimental. Anaerobic metabolism is not sustainable for long periods of time, and this may push the skate to move into new areas of the Harbour, or shallower depths where DO is higher. Given the limited habitat of this species, loss of preferred habitat may have adverse effects on the conservation of this endangered species.
